# Use of an Automated Impactor for Total Hip Arthroplasty Does Not Increase Surgeon Noise Exposure Over Manual Malleting Technique

**DOI:** 10.1016/j.artd.2026.102085

**Published:** 2026-07-09

**Authors:** Aaron M. Davidson, Jonathan Brutti, Zachary Grand, Charles M. Lawrie

**Affiliations:** a Florida International University Herbert Wertheim College of Medicine, Miami, FL, USA; b Baptist Health Orthopedic Institute, Coral Gables, FL, USA

**Keywords:** Total hip arthroplasty, Intraoperative noise, Sound exposure, Automated impaction, Occupational hearing, Acoustic measurement

## Abstract

**Background:**

Total hip arthroplasty (THA) is associated with high intraoperative noise exposure, raising concerns for occupational hearing risk. Automated impaction systems are increasingly used, but their effect on sound levels compared to traditional manual techniques remains unclear. This study evaluated intraoperative sound exposure during THA using automated vs manual impaction.

**Methods:**

In this observational study, intraoperative sound measurements were collected during primary THA via a direct anterior approach at a single institution. A calibrated external microphone positioned near the surgeon’s hearing zone recorded sound during a standardized 180-second impaction interval. Primary outcomes included peak sound level (LApeak, dB), average continuous sound level (LAeq, dB), and total sound exposure (dB-seconds). Cases were grouped by impaction method (automated vs manual). Independent *t*-tests compared sound metrics, and Pearson correlation assessed associations with implant size.

**Results:**

Twenty cases were analyzed (10 automated, 10 manual). No significant differences were observed between automated and manual impaction for LApeak (95.22 vs 95.24 dB, *P* = .975), LAeq (84.36 vs 84.23 dB, *P* = .888), or total sound exposure (15,130.5 vs 15,097.4 dB-seconds, *P* = .848). Implant size and laterality were not associated with sound measurements. Strong correlations were observed between LAeq and total sound exposure (*r* = 0.981, *P* < .001).

**Conclusions:**

Automated impaction did not significantly alter intraoperative sound exposure compared to manual techniques. Noise levels were consistent across procedural variables, suggesting that acoustic burden is inherent to THA. Procedure-level strategies may help mitigate occupational noise exposure.

## Introduction

The orthopaedic operating room (OR) is well established as one of the noisiest environments in surgical practice, with recorded levels routinely exceeding the National Institute for Occupational Safety and Health (NIOSH) thresholds of 85 dB(A) for time-weighted average exposure and 140 dB(C) for peak sound [1]. Total hip arthroplasty (THA) is a particularly loud procedure, as the use of robotic platforms and powered instrumentation often results in noise levels that exceed legally permissible occupational exposure limits [1]. However, the existing literature remains mixed regarding the magnitude and consistency of noise exposure during hip arthroplasty, warranting further investigation into whether THA is truly acoustically hazardous.

Among the operative steps comprising THA, femoral broaching is regarded as the noisiest, given its dependence on repeated mallet strikes [2]. Automated impaction systems are designed to deliver controlled, powered impaction during femoral preparation and component insertion, serving as an alternative to traditional manual mallet techniques. These devices utilize mechanically or pneumatically driven repetitive impaction to generate consistent force transmission while reducing the physical demands associated with repeated manual mallet strikes [3,4]. Proposed advantages include improved impaction consistency, reduced surgeon fatigue, enhanced ergonomics, and potentially improved implant seating accuracy [3,4]. As automated impactors gain traction in clinical practice, understanding their effect on intraoperative noise levels compared to traditional manual techniques becomes an important consideration for occupational safety.

**Figure 1:**
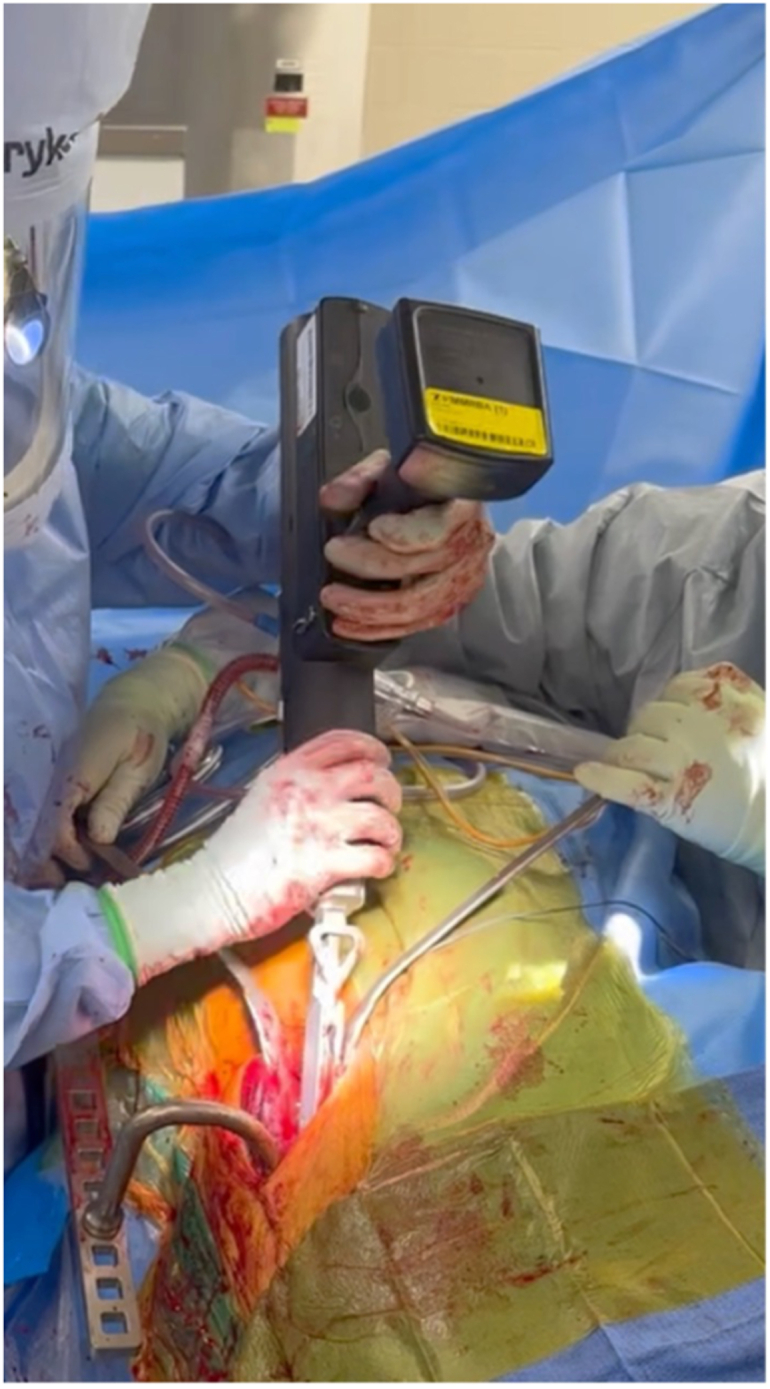
Intraoperative use of the HAMMR automated impaction device during femoral component insertion in total hip arthroplasty. The device delivers controlled, powered impaction as an alternative to traditional manual mallet techniques.

Although noise levels in arthroplasty surgery have been documented, the ongoing emergence of novel robotic platforms and powered instrumentation represents an ever-evolving operative landscape whose acoustic profile remains to be fully defined. As these systems continue to gain widespread adoption across orthopaedic subspecialties, assessing their associated noise exposure is critical to mitigating the risk of noise-induced hearing loss (NIHL) among surgical teams and OR staff [5]. The purpose of this study was to compare intraoperative sound exposure during THA between the HAMMR (Zimmer Biomet, Warsaw, IN, USA, Fig. 1) automated impaction system and traditional manual mallet impaction. We hypothesized that automated impaction would not significantly increase intraoperative sound exposure compared to manual mallet impaction.

## Materials and methods

Institutional review board approval was obtained prior to study commencement. This observational study evaluated intraoperative sound exposure during primary THA performed via a direct anterior approach at a single institution.

**Figure 2:**
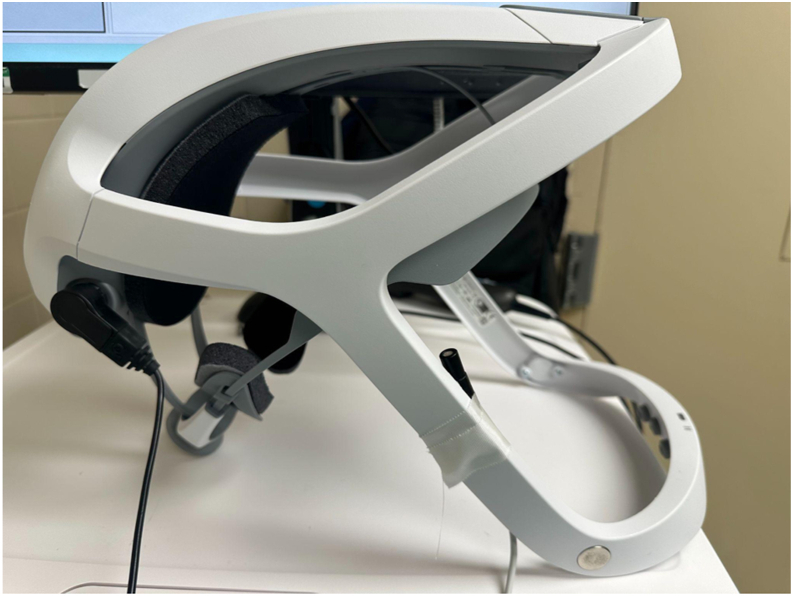
Positioning of the Dayton Audio iMM-6C calibrated external microphone secured to the surgeon’s helmet adjacent to the hearing zone within the surgical hood for intraoperative sound collection during total hip arthroplasty.

Sound measurements were collected during THA procedures using the Dayton Audio iMM-6C calibrated external microphone (Dayton Audio, Springboro, OH, USA) positioned adjacent to the surgeon’s hearing zone and affixed to the surgeon’s helmet within the surgical hood, demonstrated in Figure 2, to best measure the sound actually reaching the surgeon’s ear (Fig. 2). The device was connected to a mobile smartphone, and recording was performed using the app AudioTools (Studio 6 Digital LLC, Ventura, CA, USA), and acoustic data were continuously captured over a standardized 180-second interval corresponding to the beginning and end of femoral component impaction to ensure consistent exposure windows across cases. A standardized 180-second recording interval was selected to isolate the acoustic characteristics of the impaction phase independent of overall procedural efficiency. Standardization of recording duration also allowed cumulative sound exposure (dB-seconds) to be compared uniformly across procedures while minimizing variability introduced by pauses between sequential femoral broaches and time spent exchanging implant sizes during femoral preparation. To further reduce procedural variability, the HAMMR device was utilized by an experienced user with prior experience exceeding 100 cases, and the device was maintained at setting 2 for all broaching cases to ensure consistency across procedures.

Primary sound metrics included peak sound level (LApeak, dB), average continuous sound level (LAeq, dB), and total sound exposure (dB-seconds). LApeak and LAeq were recorded directly from the sound analysis application, while total sound exposure was calculated as the area under the curve of the sound intensity-time graph. All measurements were A-weighted to reflect human auditory perception. Cases were grouped by impaction method and compared automated impaction with the HAMMR device against manual mallet impaction. Additional variables collected included final implant size and laterality (left vs right).

Descriptive statistics were calculated for all variables and are reported as mean ± standard deviation. Independent samples *t*-tests were used to compare LApeak, LAeq, and total sound exposure between impaction methods and by laterality. Pearson correlation analysis was performed to assess the association between implant size and each sound metric, as well as interrelationships between sound measurements. Statistical significance was defined as *P* < .05. Statistical analyses were conducted with IBM SPSS Statistics (IBM Corp., Armonk, NY, USA).

## Results

A total of 20 THA cases were included, with 10 cases performed using the HAMMR device and 10 using manual (mallet) impaction. There were no missing data across variables.

**Figure 3:**
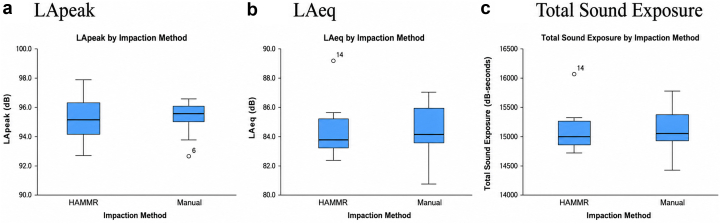
Box-and-whisker plots comparing intraoperative sound measurements between automated (HAMMR) and manual impaction. (a) LApeak (dB), (b) LAeq (dB), and (c) total sound exposure (dB-seconds). The central line represents the median, boxes represent the interquartile range, whiskers represent the range, and points indicate outliers. No significant differences were observed between groups for any sound metric.

There were no significant differences in sound measurements between HAMMR and manual impaction (Table 1, Fig. 3). Mean LApeak was 95.22 ± 1.66 dB in the HAMMR group and 95.24 ± 1.13 dB in the manual group (*P* = .975). Mean LAeq was 84.36 ± 2.02 dB for HAMMR and 84.23 ± 2.04 dB for manual impaction (*P* = .888). Total sound exposure was 15,130.5 ± 359.5 dB-seconds for HAMMR compared to 15,097.4 ± 399.1 dB-seconds for manual impaction (*P* = .848).

**Table 1:** Sound measurements by impaction method.

Outcome	HAMMR (n = 10)	Manual (n = 10)	Mean difference (95% CI)	*P* value
LApeak (dB)	95.22 ± 1.66	95.24 ± 1.13	−0.02 (−1.28 to 1.24)	.975
LAeq (dB)	84.36 ± 2.02	84.23 ± 2.04	0.13 (−1.78 to 2.04)	.888
Total exposure (dB·s)	15,130.5 ± 359.5	15,097.4 ± 399.1	33.1 (−307.5 to 373.7)	.848

CI, confidence interval; dB, decibels; dB·s, decibel-seconds; LApeak, A-weighted peak sound level; LAeq, A-weighted equivalent continuous sound level. Values are presented as mean ± standard deviation.

Final implant size was not significantly correlated with any sound measurement (Table 2). There was no significant correlation between implant size and LApeak (*r* = −0.341, *P* = .141), LAeq (*r* = 0.091, *P* = .702), or total sound exposure (*r* = 0.083, *P* = .729).

**Table 2:** Sound measurements by laterality.

Outcome	Left (n = 10)	Right (n = 10)	Mean difference (95% CI)	*P* value
LApeak (dB)	95.52 ± 1.52	94.94 ± 1.24	0.58 (−0.72 to 1.88)	.362
LAeq (dB)	84.89 ± 2.07	83.70 ± 1.78	1.19 (−0.60 to 2.98)	.185
Total exposure (dB·s)	15,220.0 ± 394.0	15,007.9 ± 330.0	212.1 (−121.6 to 545.8)	.208

CI, confidence interval; dB, decibels; dB·s, decibel-seconds; LApeak, A-weighted peak sound level; LAeq, A-weighted equivalent continuous sound level. Values are presented as mean ± standard deviation.

Laterality (left vs right) was not significantly associated with differences in sound measurements (Table 3). Mean LApeak was 95.52 ± 1.52 dB for left-sided cases and 94.94 ± 1.24 dB for right-sided cases (*P* = .362). Mean LAeq was 84.89 ± 2.07 dB for left-sided cases and 83.70 ± 1.78 dB for right-sided cases (*P* = .185). Total sound exposure was 15,220.0 ± 394.0 for left-sided cases compared to 15,007.9 ± 330.0 for right-sided cases (*P* = .208).

**Table 3:** Correlation between implant size and sound metrics.

Outcome	Correlation coefficient (r)	95% CI	*P* value
LApeak (dB)	−0.341	−0.68 to 0.12	.141
LAeq (dB)	0.091	−0.36 to 0.51	.702
Total exposure (dB·s)	0.083	−0.37 to 0.50	.729

CI, confidence interval; dB, decibels; dB·s, decibel-seconds; LApeak, A-weighted peak sound level; LAeq, A-weighted equivalent continuous sound level. Values are presented as mean ± standard deviation.

Sound measurements were significantly correlated with one another. LApeak demonstrated a moderate positive correlation with LAeq (*r* = 0.605, *P* = .005) and total sound exposure (*r* = 0.571, *P* = .009). LAeq and total sound exposure were highly correlated (*r* = 0.981, *P* < .001), indicating substantial overlap between average and cumulative sound exposure metrics. Impaction method, implant size, and laterality were not significantly associated with intraoperative sound measurements.

## Discussion

This study found no statistically significant differences in LApeak, LAeq, or total sound exposure between automated HAMMR impaction and traditional manual mallet impaction during direct anterior approach THA. Mean LApeak values were nearly identical between groups, and mean LAeq values approximated 84 dB in both cohorts, approaching but not exceeding the NIOSH recommended exposure limit of 85 dB(A) for an 8-hour time-weighted average [1]. The practical implication of these findings is that the HAMMR system does not meaningfully alter the OR acoustic environment relative to conventional malleting. From an occupational safety standpoint, surgeons and OR staff should not consider the adoption of automated impaction to carry any incremental risk for NIHL compared to the standard technique. Importantly, both methods yielded similar near-threshold LAeq values, highlighting that impaction, whether manual or powered, represents an inherent acoustic burden of THA that warrants awareness and monitoring regardless of the instrumentation chosen.

The absence of a correlation between implant size and LApeak, or total sound exposure, and the lack of any statistically significant association between operative laterality and sound measurements, further reinforce the consistency of the THA acoustic environment across patient and procedural variables. These covariates were examined given the plausible biomechanical hypothesis that larger implants requiring greater impaction force, or operative positioning differences between left- and right-sided cases, might produce measurably different sound profiles. Notably, although left-sided cases demonstrated numerically higher values across all metrics, none reached statistical significance. The null findings suggest that the acoustic burden of THA is a procedure-level phenomenon rather than one driven by anatomical or patient-specific factors. Clinically, this means that a high-volume arthroplasty surgeon performing cases across a range of implant sizes and lateralities faces a comparably consistent noise exposure profile, which simplifies occupational risk assessment and reinforces the utility of procedure-level rather than case-specific noise monitoring strategies.

The mean LAeq values of approximately 84 dB observed in both groups of the current study fall within the lower end of the previously reported range, a finding broadly consistent with the SIREN study by Goffin et al., in which THA LAeq and peak sound pressure levels did not exceed the lower exposure action value during Mako robotic-arm-assisted cases [6]. Similarly, Slaven et al. reported that time-weighted average noise exposure during both THA and total knee arthroplasty remained below the NIOSH recommended exposure limit of 85 dB, with a daily dose percentage of approximately 2% per case, suggesting that in conventional arthroplasty practice, cumulative exposure may not consistently reach hazardous thresholds [7].

While prior studies have evaluated noise exposure associated with orthopaedic instrumentation, direct comparisons between automated impaction devices and manual mallet techniques during THA remain limited. Existing literature has largely focused on automated broaching systems, with studies demonstrating increased noise levels compared to manual techniques during THA [8]. By contrast, automated broaching in the Lutz et al. cohort produced a mean LAeq of 86.09 dB(A) and a projected noise dose of 137.74%, compared to 83.06 dB(A) and 82.02% for manual broaching, values that differ meaningfully from the near-equivalent noise levels observed in the present study for both impaction methods [8]. Other investigations evaluating automated impaction systems have reported lower sound levels compared to manual techniques, though these findings were obtained in controlled or nonclinical settings [3]. Additionally, recent reviews of commercially available automated impactors, including KINCISE, Woodpecker, orthodrive, and HAMMR, have demonstrated comparable noise profiles across devices; however, these measurements were derived from experimental models and short-duration testing rather than standardized intraoperative conditions [4].

Differences in operative environment acoustics, measurement methodology, and case volume between studies may contribute to these divergent findings. Notably, prior investigations have not incorporated comprehensive acoustic metrics such as peak sound levels, average continuous sound levels, and cumulative sound exposure within a defined intraoperative time interval. To our knowledge, this is the first study to specifically evaluate intraoperative sound exposure associated with the HAMMR automated impaction system in a clinical THA setting, providing novel, procedure-level acoustic data relevant to both surgeons and OR personnel. Prior work has also documented THA and total knee arthroplasty noise levels ranging from 74.6 to 123 dB(A) across instruments including mallets, oscillating saws, and acetabular reamers, with the large majority exceeding NIOSH-recommended limits [5], a range within which the current study's findings represent a notably favorable acoustic profile for both impaction techniques.

Adult reconstruction has been identified as carrying the highest noise dose and projected dose per case among all orthopaedic subspecialties [9], highlighting the clinical relevance of acoustic characterization in this specific surgical population. Despite this, a systematic review of the historical literature found that approximately 51% of orthopaedic personnel exhibited evidence of NIHL after adjustment for expected age-related hearing loss [10], and a more recent review confirmed that few preventive measures are regularly followed in the orthopaedic setting [11], a persistent gap that reinforces the importance of device-level acoustic characterization and the development of institutional hearing conservation frameworks.

### Limitations

This study has several limitations that should be considered when interpreting its results. The sample size of 20 cases is modest, and the study was conducted at a single institution by a single surgeon, limiting the generalizability of findings across operative environments, surgical styles, and institutional acoustics. Additionally, the study did not perform task-segmented or frequency-specific noise analysis, precluding identification of which operative steps contributed most to cumulative exposure or whether the 2 impaction methods produced distinct spectral profiles. Moreover, the use of a standardized 180-second recording interval may not fully capture differences in total impaction duration or procedural efficiency between techniques. While this approach was intentionally selected to standardize cumulative sound exposure measurements and minimize variability introduced by pauses between sequential femoral broaches and implant exchanges during femoral preparation, it may underestimate or obscure differences in overall noise exposure attributable to variations in operative workflow or impaction efficiency.

The measurement system employed in this study also warrants consideration as a limitation. Sound measurements were obtained using the Dayton Audio iMM-6C calibrated external microphone. Prior validation studies comparing this device to reference sound level meters have demonstrated minimal deviation, with reported differences of approximately 0.1–0.2 dB [12]. Although this represents a small systematic offset, it is unlikely to meaningfully affect the study’s findings, as these differences are well below clinically or occupationally significant thresholds.

These results are device specific. There are now several automated impactors on the market for THA procedures, and the results and conclusions of this study should be interpreted only in the context of the device utilized (Zimmer Biomet HAMMR).

Future studies should address these limitations through larger, multicenter designs with power calculations prospectively calibrated to detect clinically meaningful noise differences. As additional automated and robotic platforms continue to enter the arthroplasty market, systematic acoustic characterization of each new system will be essential to informing occupational safety guidelines in a rapidly evolving operative landscape. Longitudinal audiometric surveillance of OR personnel, particularly high-volume arthroplasty surgeons, represents a critical and currently underutilized tool for quantifying the real-world hearing consequences of cumulative intraoperative noise exposure and should be incorporated into future occupational health research frameworks.

## Conclusions

Automated HAMMR impaction did not result in differences in peak, average, or cumulative intraoperative sound exposure compared to traditional manual mallet impaction during THA. Sound levels were consistent across implant size and operative laterality, suggesting that intraoperative noise is primarily driven by the procedure itself rather than specific technical or patient-related factors. Although mean LAeq values approached established occupational exposure thresholds, both techniques demonstrated similar acoustic profiles, indicating that the adoption of automated impaction does not introduce additional noise-related risk. These findings support the consideration of procedure-level strategies for monitoring and mitigating noise exposure in the orthopaedic OR.

## IRB statement

This study was reviewed and approved by the institutional review board prior to study commencement. All procedures were conducted in accordance with institutional guidelines and ethical standards.

## CRediT authorship contribution statement

**Aaron M. Davidson:** Writing – review & editing, Writing – original draft, Visualization, Methodology, Investigation, Formal analysis, Data curation. **Jonathan Brutti:** Writing – review & editing, Investigation, Data curation. **Zachary Grand:** Writing – review & editing, Investigation, Data curation. **Charles M. Lawrie:** Writing – review & editing, Validation, Supervision, Project administration, Methodology, Conceptualization.

## Conflicts of interest

The authors declare there are no conflicts of interest.

For full disclosure statements refer to https://doi.org/10.1016/j.artd.2026.102085.

## Appendix A Supplementary data

Conflict of Interest Statement for Davidson

Conflict of Interest Statement for Lawrie Davidson

Conflict of Interest Statement for Brutti

Conflict of Interest Statement for Grand
